# EEG-responses to mood induction interact with seasonality and age

**DOI:** 10.3389/fpsyt.2022.950328

**Published:** 2022-08-09

**Authors:** Yvonne Höller, Sara Teresa Jónsdóttir, Anna Hjálmveig Hannesdóttir, Ragnar Pétur Ólafsson

**Affiliations:** ^1^Faculty of Psychology, University of Akureyri, Akureyri, Iceland; ^2^Faculty of Psychology, University of Iceland, Reykjavík, Iceland

**Keywords:** mood induction, seasonality, seasonal affective disorder, winter depression, EEG

## Abstract

The EEG is suggested as a potential diagnostic and prognostic biomarker for seasonal affective disorder (SAD). As a pre-clinical form of SAD, seasonality is operationalized as seasonal variation in mood, appetite, weight, sleep, energy, and socializing. Importantly, both EEG biomarkers and seasonality interact with age. Inducing sad mood to assess cognitive vulnerability was suggested to improve the predictive value of summer assessments for winter depression. However, no EEG studies have been conducted on induced sad mood in relation to seasonality, and no studies so far have controlled for age. We recorded EEG and calculated bandpower in 114 participants during rest and during induced sad mood in summer. Participants were grouped by age and based on a seasonality score as obtained with the seasonal pattern assessment questionnaire (SPAQ). Participants with high seasonality scores showed significantly larger changes in EEG power from rest to sad mood induction, specifically in the alpha frequency range (*p* = 0.027), compared to participants with low seasonality scores. Furthermore, seasonality interacted significantly with age (*p* < 0.001), with lower activity in individuals with high seasonality scores that were older than 50 years but the opposite pattern in individuals up to 50 years. Effects of sad mood induction on brain activity are related to seasonality and can therefore be consider as potential predicting biomarkers for SAD. Future studies should control for age as a confounding factor, and more studies are needed to elaborate on the characteristics of EEG biomarkers in participants above 50 years.

## 1. Introduction

Rosenthal et al. (1) were the first to describe seasonal affective disorder (SAD) as a mood disorder that is characterized by recurrent depressions that occur annually at the same time of the year. Depressive symptoms in SAD are known to occur in both summer and winter, but winter depression with remission in spring is its most common representation (2).

Prevalence of SAD is estimated to be as high as 9.7% worldwide although it has been found to vary substantially based on location (3). The condition is more common among adolescents or young adults compared to elderly individuals (4, 5). The main distinctive feature of SAD as compared to major depressive disorder is that it occurs repeatedly at the same time of the year. Early screening in summer or fall could be used to plan timely prevention before first symptoms occur. It is partly possible to predict SAD based on subjective reports about the annual seasonal changes in mood, appetite, energy, sleep, and social interaction. Given that subjectively experienced seasonal changes are an important indicator for SAD, screening instruments for SAD were developed in order to measure these changes in the form of a score for an individual's *seasonality* (1). The most commonly used tool for assessing seasonality is the Seasonal Pattern Assessment Questionnaire (SPAQ) developed by (1) for the diagnosis of SAD. Presently, the SPAQ is administered as a screening tool rather than a formal diagnosis (6) as SAD requires a clinical diagnosis based on standardized criteria, such as those found in the DSM-5 (7). The SPAQ allows to calculate a global seasonality score (GSS) which is higher when individuals indicate large fluctuations of sleep, social activity, mood, weight, appetite, and energy. The SPAQ is the most widely used questionnaire to assess seasonality; For example, a systematic review yielded that 46 out of 55 samples were examined with the SPAQ (8).

Seasonal and non-seasonal depression are supposed to share the same cognitive vulnerabilities (9–11). The level of automatic thoughts and dysfunctional assumptions in individuals with SAD are comparable to the elevated scores of patients with non-seasonal depression (9). Furthermore, patients with SAD and those with non-seasonal depression might exhibit a similar style in negative attributions (10).

People being vulnerable to the onset of depression are distinguished from non-vulnerable people only if being confronted with certain stressors (12, 13). Another study confirmed the role of cognitive vulnerabilities in combination with sad mood induction, and that dysfunctional attitudes were more severe after mood induction (11). However, no prior studies included mood induction when examining individuals with SAD.

Electroencephalographic (EEG) studies have provided promising results in identifying possible indicators of psychological states and psychiatric disorders, such as depression and the cognitive processes associated with the condition (14–16). Individuals suffering from major depression disorder were reported to show lower absolute power in the electroencephalogram (EEG) in the frontal lobe compared to healthy controls, in all frequency bands but mainly in the alpha range (17). Alpha activity has been associated with emotional experience (18), self-reflection (19), and has shown a negative relationship with cognitive function and attention (20).

Asymmetrical alpha band activity between the frontal hemispheres has been found to be a likely indicator of depression, with depressed individuals having relatively higher alpha power in the left hemisphere compared to healthy controls (21–23). The frontal lobe was also reported to respond to sad mood induction (24–26) or to predict responses to mood induction (27), but it was argued that the dispositional state, examined under resting conditions may be less conclusive than the measurement under specific emotional contexts, which allows capturing emotional responses (25).

Lowered alpha activity in the prefrontal cortex is especially thought to predict higher tendency to ruminate (28). The relevance of the frontal cortex as well as the alpha frequency range for mood disorders points to the role of cognitive control over negative thoughts. High alpha power is acknowledged to reflect active inhibition (29). Therefore, the relative enhancement of alpha power in the left hemisphere can be interpreted as reduced cortical activity. It was theorized that hypoactivation of the left frontal area leads to ruminative tendencies and consequently to negative emotional interpretation (30). The frontal cortex is also relevant for cognitive flexibility (31), which has been reported to be impaired in individuals with depression (32). It was suggested that individuals with major depressive disorders exhibit ruminative and negative automatic thoughts because being cognitively inflexible in a negative emotional context (33). Depressed individuals exhibit a tendency to pay greater attention to adverse stimuli, and this tendency was linked to an abnormal activation in the lower left frontal cortex (17, 34). Abnormalities in the activation or structure of the emotion circuit have been suggested to underlie depressive disorders, including the prefrontal cortex, anterior cingulate cortex, hippocampus, and amygdala (35).

In addition to alpha abnormalities, abnormal synchronization of theta and beta oscillations was suggested to reflect unstable states of cognitive processing, specifically of working memory in individuals with depression (36). Lower power in the alpha and theta range has been reported during mind wandering (37). Moreover, increases in the delta band are generally related to pathology such as mental slowing in dementia (38), as well as psychopathology (39).

Frontal EEG asymmetry has also been studied during induced mood states (40). The induction of sad mood is related to asymmetry as compared to the induction of euphoria (41), and the level of asymmetry is related to the level of negative affect (27). A review summarized that it is likely that the hypoactivation of the left frontal lobe in response to negative stimuli reflects a predisposition to mood disorders (40). However, several findings contradict this point of view (25, 42).

Frontal alpha asymmetry seems to be subject to seasonal variation (43). Asymmetry of spectral EEG-power in frontal and parieto-temporal networks was documented also in individuals with SAD (44–47). Patients with SAD showed lower delta, theta, and alpha activity than controls (46, 47). In contrast, in remitted patients, an increase in theta power has been noted globally compared to controls (46). A similar pattern with lower EEG power can be found in patients with depression (17). This suggests that the brain activity changes in SAD and non-seasonal depression reflecting a similar mechanism of a cognitive instability. However, the rather small samples of previous studies warrant rigorous replication of these findings. Studies in healthy controls showed that there is seasonal variation of beta and alpha power, with especially high amplitudes in summer (48, 49).

When discussing EEG studies on SAD one should critically note that SAD is more common at young age (4, 5, 50), while EEG bandpower changes with age in so far as the dominant rhythm—usually alpha—shifts to a lower frequency range (51). This shift consists typically of higher power in lower frequency ranges (delta-theta) and lower power in higher frequency ranges (alpha-beta). Since depressive states coincide with lower power in the alpha range, as well, it is necessary to consider a potential interaction between age and seasonality when examining abnormalities in brain activity related to seasonality. Frontal power asymmetry is stronger in young healthy controls as compared to individuals with major depression, but the difference diminishes and even reverses with age (52). Whether or not differences in brain activity between individuals who are vulnerable to experience SAD vs. those who don't also depend on age has not been investigated, yet. Furthermore, no EEG research has been conducted on induced sad mood in individuals who score high on seasonality indices in order to identify potential neurophysiological biomarkers for SAD. Especially studies comparing brain activity of individuals with high seasonality during remission to controls with low seasonality are rather rare. The differences found between people with and without SAD in winter-time might be due to physiological changes induced by the winter's darkness that are evident only in those individuals with high seasonality. However, it would be more useful to detect differences between people with and without high seasonality in a season with more daylight. In other words, detecting neurophysiological differences between patients with and without SAD already in summer could indicate whether predispositions exist or not. Those cognitive processes that induce sad mood might potentially distinguish between individuals with high or low seasonality even during remission and support the understanding of cognitive vulnerabilities in SAD.

The novel contribution of the present study was, thus, (1) that we examined a sample with a broad age range as compared to young participants, only, (2) we examined them during late summer/early fall instead of winter, and (3) we used a procedure to induct sad mood. With this setup we aimed to answer the following questions:

- **How is a potential interaction of age and seasonality reflected in EEG band power?** Remission states of SAD come along with higher theta power. Seasonality is more common among younger individuals, and EEG band power exhibits increase of power in slower compared to faster rhythms with age. We expect a similar effect of high seasonality and higher age on EEG band power, with higher power in lower frequency ranges (delta-theta) and reduced power in higher frequency ranges (alpha-beta).
- **How does EEG band power change during induced sad mood in people with high-seasonality?** We expect participants with high seasonality to show a larger change during the induction of sad mood in the form of a stronger broadband decrease in EEG absolute power, but especially in frontal alpha.

## 2. Methods

This data was also analyzed in a previously published study by the same authors (53), where more details on the study parts that were not analyzed in the present report can be retrieved.

### 2.1. Ethics

This study was approved by The National Bioethics Committee which confirmed our application on May 28th 2019 (study number 19-090-V1). All who worked on this study signed a non-disclosure contract. We obtained written informed consent for participation from all participants.

### 2.2. Recruitment

Recruitment was done between June and September 2019 *via* publication of a webform on the University website and by posting the link to that webform on social media. The biggest outreach was obtained by sending out invitation emails to psychology students of the University of Akureyri. Psychology students could use the participation certificate obtained after completing the study as a compensation for physical attendance at a hands-on session in a seminar. Overall, 23 psychology students participated in the study. Inclusion criteria were a minimum age of 18 along with a sound mind and enough judgement to give informed consent. Proficiency in Icelandic was a requirement for participation in the study as all materials used were in Icelandic. Thus, we excluded all participants that did not speak Icelandic fluently. The study at hand was part of a more extensive project, where participants were required to complete online-follow up surveys. For completing all phases of the study, participants were offered a 4000 ISK gift certificate.

### 2.3. Questionnaires

For the purpose of assessing mood and behavioral change according to the seasons participants completed the SPAQ (1). The version used in this study was validated in Iceland against a diagnostic clinical interview with a resulting sensitivity of 94%, a specificity of 73% and a combined positive predictive value of 45% for SAD and subsyndromal SAD (54). The questionnaire has proven to be an effective screening tool for SAD, is internal consistent (α = 0.74–0.81), reliable (with a test-retest reliability of 0.76 at an interval of 2 months), and widely used in SAD research compared to similar measures to which it was compared, such as the inventory for seasonal variation (55). However, a high seasonality score does not equal a diagnosis of SAD according to the DSM-5 criteria (56). To obtain participants' global seasonality scores (GSS), we calculated the sum of the global seasonality questions in the SPAQ. As a non-clinical estimate for SAD, we grouped participants into low- and high seasonality by means of the Kasper criteria (57), according to which SAD is defined as a GSS above 10. A larger score indicates that the individuals report to be more likely to experience seasonal variation in mood, energy, weight, appetite, sleep, and social activity. We did not further distinguish between SAD and subsyndromal SAD, as the ability of the SPAQ to differentiate between these two subsamples was found to be rather poor (54).

In addition, participants completed the Depression Anxiety Stress Scale (DASS, 88), Patient Health Questionnaire (PHQ, 87), and Bergen Insomnia Scale (BIS, 58). The BIS can be used to measure insomnia according to the formal and clinical diagnostic criteria (DSM- IV-TR) and consists of six items. The first three items assess to sleep onset, sleep maintenance, and early morning awakening. The last three items ask about not feeling adequately rested, experiencing daytime impairment, and dissatisfaction with sleep. The scale can be scored with a total composite score ranging from 0 to 42. The original scale was validated by (58) and the Icelandic version had been translated and validated previously (50).

Participants were also asked about their age, gender, education, handedness, and first language.

Mood was measured on a visual analog scale as relation between the indicated position to the total length of the bar, measured in millimeters. The total length was 150 mm with arrows indicating increase strength of mood from the middle of the scale with the left arrow indicating sad mood and the right arrow indicating happy mood. This tool was used previously in similar sad mood induction task studies (59, 60). Cognitive flexibility was measured as reaction time difference between congruent and incongruent conditions in the Stroop task. For this purpose we subtracted the mean of reaction time in congruent trials from the mean of reaction time in incongruent trials, and we grouped participants by a median-split.

### 2.4. Procedure

Measurements were performed at the EEG-laboratory of the University of Akureyri between end of July and beginning of October 2019. Experimenters were present in the laboratory throughout the whole procedure. After completion of informed consent, participants answered all questionnaires mentioned in Section 2.3 and the EEG-cap was mounted.

The first two conditions recorded were at rest for 3 min with eyes open and 3 min with eyes closed, with the screen of the stimulus computer turned off and dimmed light. Subsequently participants performed an emotional pictures memory task, which was not used for the present study.

The next condition was a Stroop task where participants were asked to indicate the font color of words displayed on the stimulus computer by pressing a correspondingly colored key on a keyboard where the color of the font corresponded to the word. There were 105 congruent trials and 210 incongruent trials presented in a randomized order, with an inter-trial interval of 1 s + a jitter of 0-10 screen flip intervals during which a central fixation cross was presented.

In the final condition participants first received a printed three part form containing the questions about current mood in the form of the visual analog scale and measurement of induced rumination that was not used for the purpose of the present study. Time spent on answering the written questions on mood and rumination did not count toward the indicated time-periods. All instructions were given verbally through headphones or on the screen of the stimulus computer. Firstly, participants rated their current mood on the visual analog scale and state rumination *via* a short state rumination inventory questionnaire. Next, participants listened to an 8 min musical piece, thought to evoke temporary sadness or dysphoria. Participants were asked to freely experience any emotions they might feel. We used a musical excerpt from Prokofiev's “Russia Under the Mongolian Yoke”, remastered at half speed. This has been used and found to be effective in inducing a transient dysphoric mood in previous research on cognitive vulnerability to depression (60–62).

Immediately after the music had finished, participants rated their current mood again on the visual analog scale. Then, they were then instructed to wait for 5 min for a challenging cognitive task. From this 5 min free thinking period we extracted the first 3 min for EEG analysis. However, no cognitive task followed but the waiting period served as a free contemplation time in anticipation of a task. This instruction for the free thinking period is a new procedure and was chosen in order to try to keep participants focused on themselves and the upcoming experiment and to counter that their mind starts to wander about other issues such as their surrounding. Finally, participants rated their current mood for a third time on the visual analog scale and completed the rumination state evaluation *via* the short state rumination questionnaire for a second time. After this, participants were informed that no difficult task would follow and that the study was completed.

To sum up, for the present study, we used the EEG data recorded during 3 min rest with eyes open condition and during the first 3 min of the free-thinking period after listening to the sad music, which was intended to induce sad mood. Due to the emotional pictures memory task and Stroop task that were conduced in between these two conditions, the free-thinking period followed about half an hour after the resting condition. We did not control for drowsiness/wakefulness but participants were asked to keep their eyes open during both of these periods, to keep the overall background condition comparable and to prevent participants from falling asleep.

### 2.5. EEG recording and analysis

EEG data was gathered using BrainVision BrainAmp Recorder and Amplifier (Brain Products GmbH, Gilching, Germany) and digitized at a sampling rate of 256 Hz. Recording was conducted using 32 electrodes (Fp1, Fp2, F3, F4, C3, C4, P3, P4, O1, O2, F7, F8, T7, T8, P7, P8, Fz, Cz, Pz, FC1, FC2, CP1, CP2, FC5, FC6, CP5, CP6, FT9, FT10, TP9, TP10) referenced to FCz and grounded at AFz. One additional electrode served as lower vertical electrooculogram. Electrode positioning was in accordance with the standardized international 10-20 system by using an EasyCap. Electrodes were filled with electrolyte containing a mild abrasive (OneStep Abrasive Plus) in order to achieve low impedance of <10kΩ in all channels.

We analyzed EEG-data from 3 min rest with eyes open condition and the first 3 min of the free-thinking period after listening to the sad music. EEG-data was pre-processed using BrainVision Analyzer (Brain Products GmbH, Gilching, Germany). Data was filtered from 0.5 to 30 Hz with zero-phase shift Butterworth filters. Then, re-referencing was performed by averaging the activity of all electrodes and subtracting this mean from all other channels. Next, an independent component analysis (ICA, infomax restricted) was performed in order to automatically remove eye-blink artifacts. The vertical lower oculogram was used as a vertical activity channel. The whole recording epochs per condition were fed into the algorithm, i.e., at least 3 min per condition as the shortest condition was 3 min of rest with eyes open or closed. The ICA (63) is an algorithm that separates the EEG signals into the same number of temporally maximally independent component time courses. The ocular component represents eye movements and blinks and, thus, has a characteristic pattern in time and topography, which is used for the automatic selection of the component. To this end, the ocular correction ICA first performed a blink marker placement by searching the oculogram channel for blinks and marking them according to the mean slope algorithm (64). For identification of components related to the vertical electrooculogram only the time intervals that are identified by this algorithm as blinks are used. Then, the share of each ICA component in the variance of the selected ocular channel activation was calculated, which was then excluded from back-projection. As a last pre-processing step, a raw data inspection was done by applying the standard thresholds as implemented in Brain Vision Analyzer, in order to automatically identify and exclude movement and muscle artifacts: check gradient: the maximal allowed voltage step is 50 microvolt/ms; check difference: the maximal allowed difference of values in intervals of 200 ms: 200 microvolt; lowest activity allowed in 100 ms intervals is 0.5 microvolts; bad events were marked ±20 0 m around the identified artifacts. The two 3 min recordings were divided into 2 s segments. For each segment, we calculated the Fast Fourier Transform (FFT). The FFT of all segments that were not marked as containing artifacts were averaged for each participant, separately for the two conditions rest and sad mood induction. From the average FFT we extracted average band power in the delta (1–4 Hz), theta (5–7 Hz), alpha (8–13 Hz), and beta (14–30 Hz) range for statistical analysis. EEG channels were grouped for lobes and hemisphere for the purpose of conducting an analysis of variance with factors lobe and hemisphere, but used individually for *post-hoc* illustrations of results. When more than one electrode was recorded for one such region, averaging was performed. These regions were frontal-left (Fp1, F3, F7), frontal right (Fp2, F4, F8), central left (C3), central right (C4), parietal left (P3, P7), parietal right (P4, P8), temporal left (T7), temporal right (T8), occipital left (O1), and occipital right (O2).

### 2.6. Statistical analysis

For comparing psychometric characteristics between the group with low and high seasonality we used non-parametric tests because all data was ordinal, and thus, no parametric tests are allowed. Therefore, we calculated 7 non-parametric Mann–Whitney *U*-tests. The results were interpreted at the Bonferroni-corrected level of significance, that is, *p* < 0.007.

We tested for behavioral effects of mood induction with non-parametric repeated measures ANOVA with a parametric bootstrap (65) for the change in mood. When testing for interactions between seasonality and condition we also controlled for age and cognitive flexibility (31–33). Cognitive flexibility was added as a grouping variable according to a median split of reaction time increase between the congruent and incongruent color naming condition in a Stroop task. Age was also used as a grouping variable with people being up to 50 years vs. those who were older. Because there were only 18 participants older than 50, the additional combination with low vs. high seasonality and cognitive flexibility left too few participants in the subcategories of older participants with higher seasonality and low vs. high cognitive flexibility. Therefore, two separate analyses of variance were conducted, one with seasonality as a grouping factor and one with age as a grouping factor. Therefore, results were interpreted at the Bonferroni-corrected level of significance, that is *p* < 0.025.

The EEG data was evaluated using a semi-parametric repeated measures ANOVA with a parametric bootstrap (65) with between-subjects factor GSS (low vs. high seasonality), and within subjects factors hemisphere (left vs. right), lobe (frontal, central, temporal, parietal, and occipital), frequency (delta, theta, alpha, and beta), and condition (rest vs. sad mood induction). Additionally, we included factor age into the analysis, since EEG changes with age. We divided the sample into participants up to 50 years and participants older than 50 years, because prior EEG-research on SAD investigated only subjects up to age 50 (46) and because prior research indicates that 50 is a significant turning point in EEG signal properties (66).

We chose a semi-parametric repeated measures ANOVA that only requires metric data, but allows for non-normality and variance heterogeneity (65). This method is implemented in the function RM of the R-package MANOVA.RM (67). We used it with the parametric bootstrap with 1000 iterations. The parametric bootstrap showed the most favorable performance in unbalanced designs like in our case where low seasonality is much more frequent than high seasonality and, additionally, the older subgroup shows fewer cases of high seasonality than the younger subgroup (65). The method was shown to be robust and advantageous in unbalanced designs and for a large number of factors as well as EEG data previously because these data typically violate assumptions of classical methods (65).

For *post-hoc* analyses of significant interactions and effects we used *z*-values from Wilcoxon rank sum test or signed rank test for creating topographic plots of the data.

## 3. Results

### 3.1. Sample

A total of 119 participants were recruited for this study. For the statistical analysis, 3 participants were excluded due to missing data in the EEG recording. Furthermore, 2 participants (nr. 2 and nr. 76) did not complete the SPAQ and were therefore excluded. The final sample consisted of 114 participants, 10 tested in second half of July, 28 in August, 72 in September, and 3 in the first week of October. It should be noted that the weather in the Icelandic summer between end of July and beginning of October is comparable to the central-European fall between September and November.

Participants' age ranged from 18 to 66 years with the average age of 33.75 (*SD*=13.43) years. The sample consisted of 92 women and 22 men. The odds ratio for gender to suffer from SAD is 1.8 according to (68) justifying an overrepresentation of female participants. However, in our sample there were even more women to men, due to the recruitment among psychology students which are about 90% women, and also the increased availability of female voluntary participants. Eight participants were left handed, and three participants indicated to have no preference for left or right.

In the sample, 9.91% had completed primary education, only, 55.86% had higher education entrance qualification, 6.31% had learned a trade, 18.92% had completed undergraduate education at a University, and 9.01% had completed master or doctoral level education at a University. The native language was Icelandic in 95% of the sample, however, all participants were fluent in Icelandic.

We found that 57.66% reported taking any kind of medication regularly. However, this included also oral contraceptives, which explained major part of this large proportion. Furthermore, 25.44% consumed tobacco regularly. It should be noted that this consumption includes not only smoking, but also vapes, e-cigarettes, and chewing tobacco. While 20.72% reported never drinking alcohol at all, 36.94% reported drinking once a month or less, 36.94% reported drinking two to four times a month and only 5.4% reported drinking two to three times a week or more frequent drinking. Participants reported drinking on average 2.83 cups of coffee or caffeinated drinks per day (median = 2; SD = 2.54). The hours of sleep in the night before the experiment were on average 7.10 h (median = 7; SD = 1.70).

### 3.2. Psychometric and grouping data

Participants were divided into two groups based on an estimated likelihood of them experiencing mild to moderate seasonal affective disorder, measured by the GSS obtained from the SPAQ. According to (68) and (3), which used the same SPAQ questionnaire in Icelandic as we did in our study and where SAD was determined by means of the Kasper criteria (57) with a GSS score of 11 or higher. Therefore, in our study a score of ≤ 10 was categorized as a low seasonality score and a score of >10 as a high-seasonality score. Out of 114 participants that completed the SPAQ, 37 had a high GSS and 77 had a low GSS.

Descriptive statistics for the psychometric scales, separately for the two groups as well as results from Mann–Whitney *U*-tests comparing the two samples are shown in Table 1.

**Table 1:** Psychometric characteristics of the sample separately for the low and high-seasonality groups.

	**GSS** ≤ **10**		**GSS**>**10**		***U*** **-Test**	
**Scale**	**Mean**	***SD***	**Mean**	***SD***	***z***	***p***
age	36.43	14.07	29.34	10.30	2.48	0.013
GSS	4.53	2.87	14.11	2.64	−8.64	**<0.001**
BIS	13.36	8.42	21.49	7.77	−4.68	**<0.001**
PHQ	4.97	3.67	10.80	4.46	−6.00	**<0.001**
DASS depression	2.97	3.68	5.72	4.05	−3.96	**<0.001**
DASS anxiety	2.18	2.60	6.49	4.65	−5.07	**<0.001**
DASS stress	5.40	4.15	9.59	4.20	−4.45	**<0.001**

GSS, global seasonality score; DASS, depression/anxiety/stress scale.

PHQ, patient health questionnaire; BIS, Bergen insomnia scale.

Bold p-values: significant at Bonferroni-corrected level p < 0.007.

### 3.3. Behavioral responses to mood induction

The experiment involved a mood induction phase (listening to sad music) and a free thinking period. We measured mood before (t1) and after (t2) listening to sad music and after the free thinking period (t3). Mood ratings and cognitive flexibility measures in the form of reaction times for congruent and incongruent conditions in the Stroop task are given in Table 2. There were 17 participants who did show an absolute increase of mood according to the visual analog scale. However, given the inaccuracy of such a scale, we investigated how many actually meant that their mood did not change. Out of these 17 participants, 6 had a change smaller than 5 mm, 5 had a change between 5 and 10 mm, 2 had a change between 10 and 20 mm, and only 4 had a change larger than 20 mm. Thus, there were 4 with an atypical change in mood during mood induction. Among them, only 2 showed more positive mood also at t3.

**Table 2:** Mood induction reports and cognitive flexibility separately for the low and high-seasonality groups.

	**GSS** ≤ **10**		**GSS**>**10**	
**Scale**	**Mean**	***SD***	**Mean**	***SD***
Mood t1	113.43	25.65	92.93	24.90
Mood t2	80.23	38.44	68.69	36.43
Mood t3	98.24	32.85	74.08	32.65
RT congruent	778	170	749	187
RT incongruent	847	193	811	220

t1, t2, t3: time points during experiment.

RT: reaction time in milliseconds.

According to the ANOVA, there was a significant effect of time, i.e., an) induction of sad mood from t1 to t2 and t3 [*F*_(1.90,*Inf*)_ = 6.38; *p* = 0.006] as well as a significant group effect, thus, lower mood in participants with higher seasonality [*F*_(1,364.85)_ = 13.30; *p* < 0.001]. Furthermore, an interaction indicated that there was a significantly stronger effect of mood induction in the group with higher seasonality [*F*_(1.9,*Inf*)_ = 8.83; *p* = 0.001], but there was no three-way interaction with cognitive flexibility. However, there was a significant interaction between cognitive flexibility and mood induction [*F*_(1.89,*Inf*)_ = 4.85; *p* = 0.008], such that participants with a smaller increase in reaction time in the Stroop task responded more intensely to mood induction. Younger participants showed lower mood [*F*_(1,115.87)_ = 7.05; *p* = 0.022]. The interaction between age and cognitive flexibility was not significant after Bonferroni-correction for multiple comparisons.

### 3.4. Brain responses to mood induction

In order to test the interaction of effects between seasonality (two groups by GSS, low and high) and age(<51 and older) on brain activity at rest and during mood induction (factor condition) we performed a semi-parametric ANOVA. Furthermore, the model included EEG-frequency band, hemisphere, and brain lobe as factors. The results of the semi-parametric ANOVA with *p* < 0.1 are shownin Table 3.

**Table 3:** ANOVA-type semi-parametric analysis of variance with parametric bootstrap on EEG measures for factors condition (within: rest vs. after mood induction), seasonality (between: GSS low vs. high), age (between: young vs. old), hemisphere (within: left vs. right), lobe (within: frontal, central, temporal, parietal, occipital), frequency (within: delta, theta, alpha, beta).

**Factor**	**ATS**	**df1**	**df2**	***p*-value**
GSS	11.05	1	693.03	<0.001
Age	96.13	1	693.03	<0.001
GSS×age	30.02	1	693.03	<0.001
Lobe	87.42	3.24	693.03	<0.001
Age×lobe	4.14	3.24	693.03	.002
Frequency	1218.38	1.97	Inf	<0.001
GSS×frequency	10.51	1.97	Inf	<0.001
Age×frequency	29.79	1.97	Inf	<0.001
GSS×age×frequency	6.72	1.97	Inf	<0.001
Lobe×frequency	39.05	5.85	Inf	<0.001
Age×lobe×frequency	1.40	5.85	Inf	.018
Condition	185.17	1	Inf	<0.001
GSS×condition	3.23	1	Inf	.089
Age×condition	4.70	1	Inf	.039
Lobe×condition	46.34	2.53	Inf	<0.001
Frequency×condition	239.77	2.23	Inf	<0.001
GSS×frequency×condition	3.08	2.23	Inf	.027
Age×frequency×condition	4.73	2.23	Inf	<0.001
Lobe×frequency×condition	18.73	5.54	Inf	<0.001

Only effects with p < 0.1 are shown.

ATS, ANOVA-type statistic; df, degrees of freedom; GSS, grouping by global seasonality score; condition, rest vs. mood induction.

**Figure 1:**
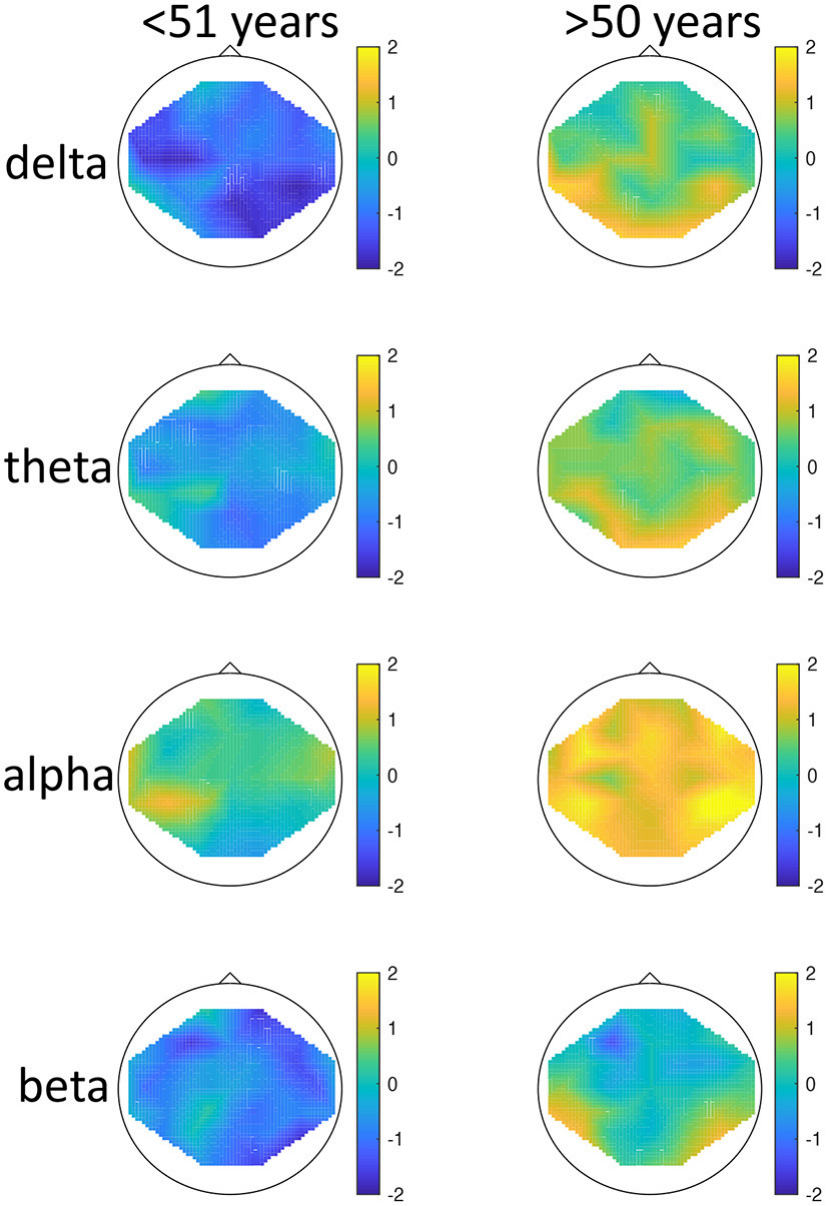
Topographical maps of activity distribution: colors represent difference between participants with low and high global seasonality score, indicated by approximated *z*-values from a Wilcoxon rank sum test, for frequency ranges from **top** to **bottom**: delta, theta, alpha, beta; and for age groups <51 years **(left)** and >50 years **(right)**. Negative *z*-values indicate lower activity in the low- compared to high-seasonality group, positive *z*-values indicate higher activity in the low- compared to high-seasonality group.

There was a significant effect of seasonality, such that high-seasonality participants had a higher overall average power in EEG oscillations (mean = 0.51; *SD* = 0.13) than low-seasonality participants (mean = 0.50; *SD* = 0.12). This difference was calculated across all frequency ranges and regions and is therefore rather small, as there were frequency ranges with opposing differences and the difference depended additionally on age (see Figure 1). The older group had a lower overall average EEG activity (mean = 0.44; *SD* = 0.17) than younger participants (mean = 0.50; *SD* = 0.12). There was a significant interaction between age, seasonality, and frequency (Figure 1). In the younger group, high-seasonality participants exhibited larger power (average between rest and mood induction) in the delta, theta and beta frequency band but lower power in the alpha range, especially in the left temporo-parietal region. In the older participant group, high-seasonality participants exhibited lower power in all frequency ranges, especially in the alpha range.

There was also a significant interaction between seasonality and frequency, such that in all frequency bands the activity was higher in high-seasonality participants except for the alpha band, where the activity was lower in that group (see Figure 1). The higher power in high-seasonality participants was most pronounced in the delta band, irrespective of condition, while higher power in low-seasonality participants in the alpha band was most pronounced during mood induction.

**Figure 2:**
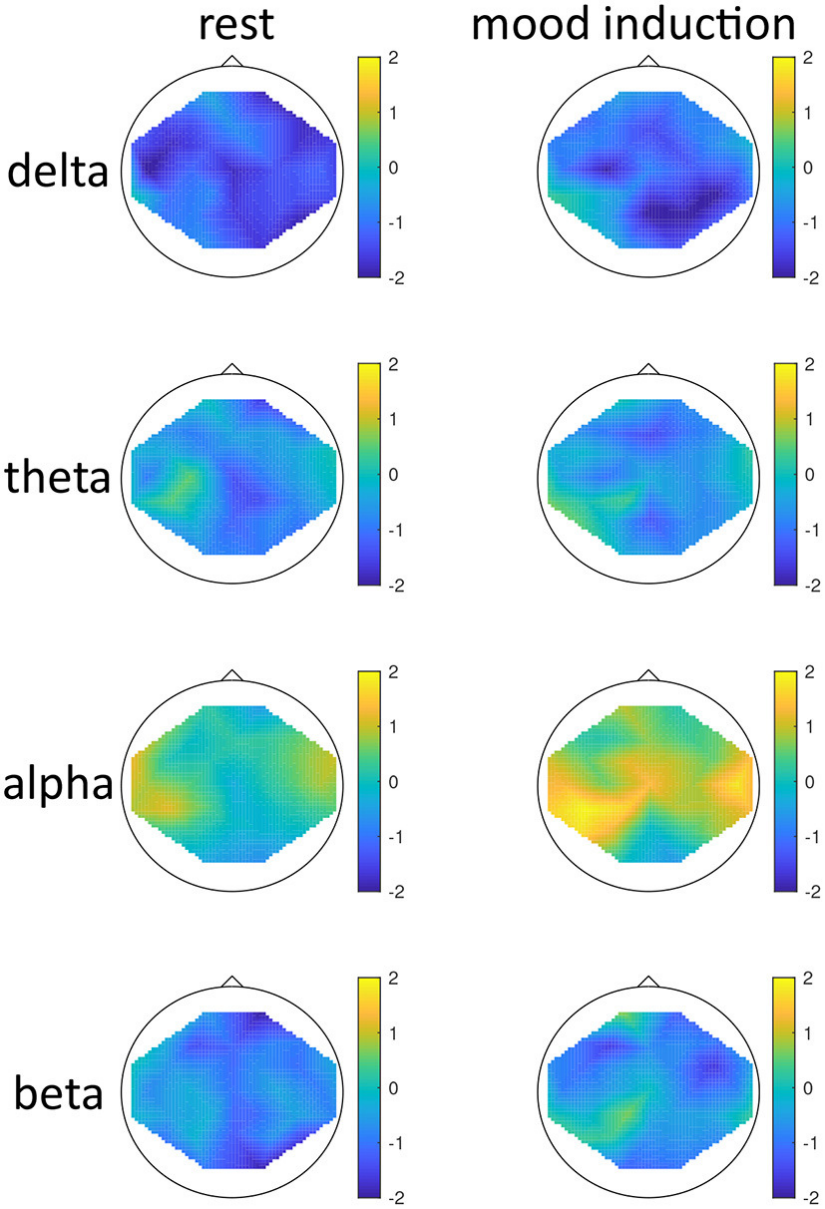
Topographical maps of activity distribution: colors represent difference between participants with low and high global seasonality score, indicated by approximated *z*-values from a Wilcoxon rank sum test, for frequency ranges from **top** to **bottom**: delta, theta, alpha, beta; and for conditions rest **(left)** and after mood induction, during the free-thinking period **(right)**. Negative *z*-values indicate lower activity in the low- compared to high-seasonality group, positive *z*-values indicate higher activity in the low- compared to high-seasonality group.

**Figure 3:**
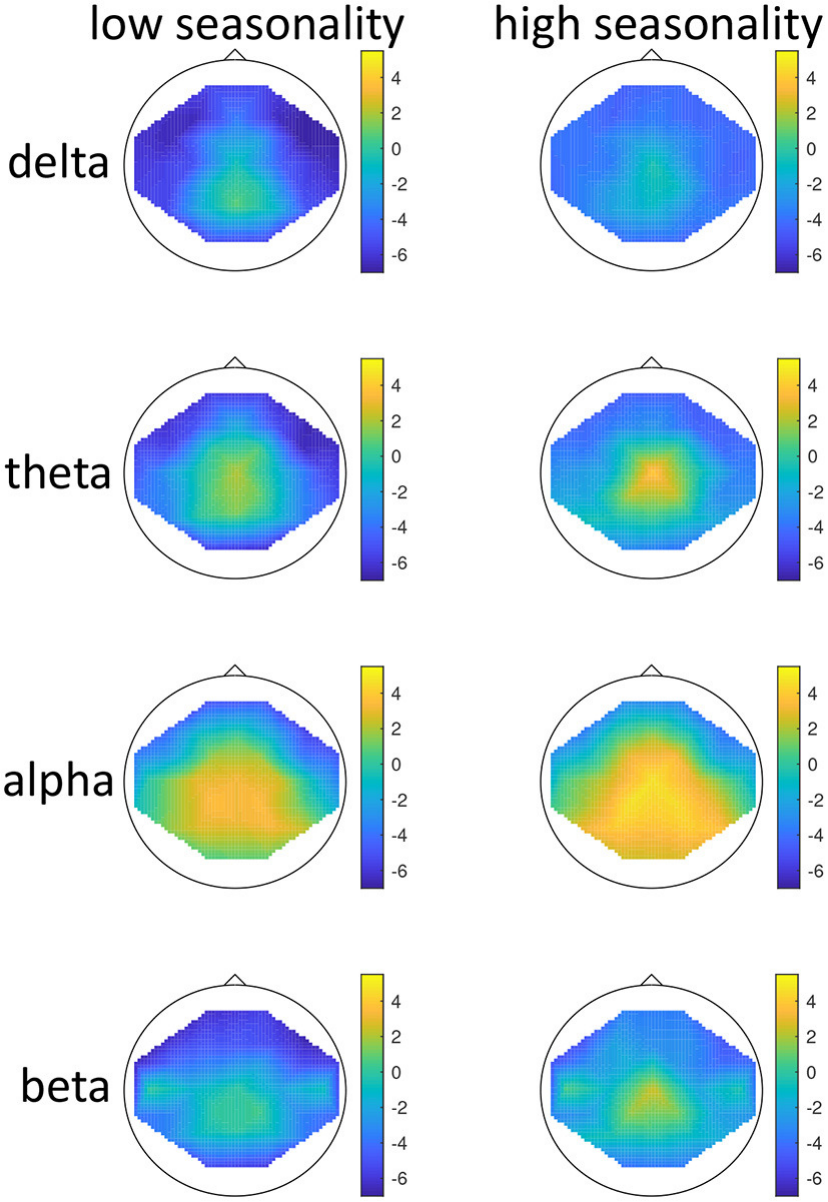
Topographical maps of activity distribution: Colors represent difference between rest and after mood induction, indicated by approximated *z*-values from a Wilcoxon signed rank test, for frequency ranges from **top** to **bottom**: delta, theta, alpha, beta; and for groups with low **(left)** and high **(right)** global seasonality score. Negative *z*-values indicate lower activity during rest compared to mood induction, positive *z*-values indicate higher activity during rest compared to mood induction.

The effect of mood induction was significant with a higher activity after induced mood than during rest, especially in the delta range and inferior frontal regions for low seasonality participants, while the largest difference was found in the alpha range for high-seasonality participants with lower activity after mood induction than during rest (see Figures 2, 3). In other words, the alpha band desynchronizes in response to mood induction, which is stronger in participants with high-seasonality. In the theta range the central region showed a desynchronization during induced sad mood while higher power was found in the frontal area.

## 4. Discussion

In this study, we examined brain activity in the delta, theta, alpha, and beta frequency bands in low and high-seasonality individuals during rest and mood induction. In addition, we controlled for age effects. Table 4 summarizes the main findings.

**Table 4:** Summary of the main findings of the study.

**Frequency range**	**Seasonality**	**Seasonality × age**	**Mood induction**
Delta	↑	↓	↑
Theta	↑	↓	↑↓
Alpha	↓	↓	↓
Beta	↑	↓	↑

↑, increased power; ↓, decreased power.

### 4.1. Brain activity, seasonality, and age

We found significant overall difference in brain activity according to seasonality, but also significant effects of age. The aging effect in the EEG was to be expected as with age, slowing of the EEG occurs, and a relative increase of delta compared to higher frequency ranges can be observed (69). Our results showed opposing directions of effects in the younger compared to the older sample. Our main interaction of age and seasonality warrants an attempt of an interpretation, i.e., younger, high-seasonality participants exhibited overall larger power, while in the older participant group, high-seasonality participants exhibited overall lower power, especially in the alpha range. Such a reversal effect that appears to be caused by age was previously documented also in patients with major depressive disorder (52) where young healthy controls showed higher frontal asymmetry in the delta, alpha, and beta frequency range, while with age, major depressive disorder patients showed larger frontal asymmetry. While the higher activity in the theta band for young SAD patients in remission was reported previously (46), nothing is known so far about effects of seasonality in the EEG of older individuals. The increased activity in the younger sample could be seen as an overcompensatory effect during summer, as during depressed states, the amplitudes of brain activity get lowered. Another possible view on this finding is supported by previous reports on higher power in the alpha and beta band in a general population in the summer (48, 49). Our finding that even higher amplitudes are found in the subsample with high seasonality score could reflect the special situation of high-seasonality individuals overreacting to the bright light conditions in the summer. Indeed, this finding was not reported in an earlier study (49) where seasonality did not interact with seasonal changes in EEG power. However, our study was conducted in Iceland with abundant amounts of daylight in the summer, whereas (49) examined participants in southern Italy (Napels), where some hours of darkness are achieved during the night. This reactivity might reverse with age because the absolute power in the alpha range decreases naturally with age. Another potential explanation for the interaction with age are changes in sleep buildup that differs between younger and older participants, as the need for sleep decreases with age. The two groups differed also significantly by quality of sleep according to BSI, which might confound these findings. Sleep disturbances in summers with long photoperiods might be more common in people who show high degrees of seasonality and might be even more common in the elderly subgroup. This interpretation is also supported by prior research which demonstrated a more rapid buildup of subjective sleepiness and EEG theta-alpha activity in patients with SAD compared to a control group (70).

Therefore, we strongly recommend controlling for age in future studies. Previous research was conduced with participants aged 30–50 years (46) or 28–55 years (47). Our study also emphasizes that, although the condition is rather rare in the elderly, further research is needed on the specific effects seasonality might have in a senior population.

### 4.2. Brain activity and SAD vs. seasonality

In contrast to studies examining active SAD (46, 47), we found increased power in all frequency bands except for the alpha band in young high-seasonality participants. Thus, our results are in line with prior reports on SAD during remission, where young patients demonstrate higher band power in the delta, theta, and alpha band compared to controls (46). EEG studies on brain activity in major depressive disorder patients have also indicated a brain-wide decrease in theta activation as well as an increase in higher frequency beta activation (71).

It is noteworthy that although we included hemisphere as a factor, there was no significant effect or interaction related to it, which is unexpected. Prior research would have suggested that our data could show an interaction between seasonality, lobe, and hemisphere, as frontal EEG asymmetry has been a hallmark of depression (21–23). But our sample was examined during remission, which might explain the lack of such an effect.

### 4.3. Brain activity during mood induction

High-seasonality individuals showed a larger and more widespread difference in the alpha range between rest and sad mood induction compared to low-seasonality individuals. Studies on rumination in major depressive disorder have established that the frontal lobe is a critical area for cognitive processes linked to depressive symptoms (14). Specifically, the induction of sad mood is supposed to evoke frontal asymmetry (40, 41). However, contrary to expectations we did not find any interaction between hemisphere and condition in our study. The relatively mild dysphoria induced by the procedure chosen might explain the lack of a clear effect in our data.

An analysis of the change in brain activity from rest to induced sad mood revealed larger frontal activity in the delta and theta frequency range during sad mood induction compared to rest.

Frontal EEG activity is greater when emotion regulation is efficient, i.e., when the response to sad mood induction is smaller (25).

In addition to enhanced delta and theta activity, we also found enhanced frontal activity in the beta range during mood induction. Beta oscillations play a crucial role in both positive and negative emotional tasks, as well as cognitive tasks (72). Increased activation of theta is thought to underlie top-down cognitive control (73), evaluation of goal directed behavior (74), and cognitive workload (75).

### 4.4. Limitations

As a result of the limited time frame of assessments during summer and location of the study in a small town with limited access to large numbers of potential participants an equal distribution of seasonality, age, gender and other demographic variables did not prove possible. Furthermore, substantial percentage of the sample consisted of university students which usually are a more homogeneous group than the general population. Especially the gender distribution was heavily biased toward women, as more women volunteered for participation. The low number of participating men also hindered us from taking gender into account as a factor in our statistical model. Unequal group sizes were compensated by the choice of statistical tests that are valid under conditions with unequal sample sizes. Nevertheless, a limitation of generalization must be considered, such as the results might be more representative for women than men. So far, sex differences were examined with respect to SAD and seasonality only in terms of the higher prevalence of seasonal symptoms among women (50), while the knowledge about interactions between sex, seasonality and EEG activity is rather limited. According to our recently published longitudinal study, sex has not predictive value when used alone or in combination with the EEG for predicting winter-time depression based on summer-time measurements (53). However, sex is known to affect EEG band activity insofar as it is possible to derive the subject's sex from EEG biomarkers (76, 77). Future studies with well-balanced samples should investigate the potential role of sex in the moderation of EEG band power interactions with seasonality.

When interpreting the results of this study, it is also important to keep in mind that the measure of seasonality is based on a screening and not a formal clinical diagnosis. SAD is a type of seasonally recurrent major depression that requires diagnosis based on clinical criteria, such as those found in DSM-5 (6). In addition, the cut-off point of GSS scores used to distinguish high and low seasonality individuals does not necessarily serve as realistic categorization of SAD but rather an indication of a possible problem (6).

Furthermore, we did not apply clinical exclusion criteria, such as prior personal history of depression, prescribed medication that is active on the central nervous system, use of stimulants, presence of psychopathology, substance abuse, history of neurological diseases such as multiple sclerosis or epilepsy, previous brain surgery or head trauma. Therefore, the data of some participants might not be fully representative for a healthy population, but could be biased. The non-clinical nature of the study and, therefore, the lack of a diagnostic interview prevented us from ruling out additional diagnoses that may be not related to seasonality or depression. Therefore, we must also consider a bias by such conditions. There are other mental disorders that follow a seasonal pattern, such as schizophrenia (78), anxiety (79), bulimia nervosa (80), and posttraumatic stress disorder (81), which might interfere with the results.

Another limitation is the choice of the mood induction procedure, where we combined verbal instructions and a sad musical piece. Although previous studies have supported this procedure (60–62), the question remains whether interindividual differences in music taste might affect the result. On the other hand, there is evidence for interindividual differences in EEG-responses to self-selected music for different purposes (i.e., activating vs. relaxing music) (82) such that letting participants choose their favorite sad music might not have led to a more homogeneous result.

Finally, this study was conducted in a cross-sectional design, while longitudinal designs are needed to confirm the relation between EEG correlates of induced sad mood in one season, i.e., summer, and depressive symptoms in the season when SAD typically occurs, i.e., winter. Such data was published recently, showing that cognitive vulnerabilities are better suited to predict winter depression, but the combination of those markers with EEG features can be advantageous (53).

### 4.5. Future directions

Recent technological developments go beyond the simple documentation of regional and frequency differences and provide automatic means for classification of brain diseases (83), for example by the use of convolutional neural networks (CNN) as a means of artificial intelligence for the classification of patients with major depressive disorder (84), which allow to determine the most information bearing regions and frequency bands (85). These methods are most useful when higher-dimensional features are chosen, such as functional connectivity measures (86). The disadvantage of CNNs is the need for a large sample size as their learning ability and generalizability depends highly on the size of the ground truth. In seasonal affective disorder, a simple support vector machine classification can be used to predict winter-time depression based on summer-time psychological vulnerabilities and EEG-features (53). The present results might add to the selection of prior knowledge for future artificial intelligence models, i.e., by adding age to the feature vector.

## 5. Conclusions

With respect to the initially posed research questions, we can draw the following conclusions:

- *How is a potential interaction of age and seasonality reflected in EEG band power?* Younger participants with high seasonality showed increased EEG power in all bands but the alpha range, while the older group with high seasonality exhibited decreased EEG power in all frequency bands. This finding emphasizes that it is important to control for age in future studies on brain activity in seasonal affective disorder.
- *How does EEG band power change during induced sad mood in people with high-seasonality?* Participants with high seasonality showed a larger difference in the alpha band with higher activity during rest compared to activity during sad mood induction possibly reflecting a breakdown of inhibition, while low seasonality participants showed a stronger frontal activity during sad mood induction across frequency ranges, possibly reflecting effective inhibition of ruminative thoughts that lead to sad mood.

Future research can make use of our results and estimate whether EEG biomarkers during induced sad mood in combination with the seasonality score in summer could serve as a predictor for SAD in winter. It is furthermore of interest whether brain activity during sad mood induction among SAD patients changes with seasons.

## Data availability statement

The datasets generated for this study can be found in Höller, Yvonne (2022), “Data for: EEG-responses to mood induction interact with seasonality”, Mendeley Data, v1 http://dx.doi.org/10.17632/7bzjts53xv.1 and the raw EEG data in Höller, Yvonne (2022), “Data for: EEG responses to mood induction”, Mendeley Data, v1, http://dx.doi.org/10.17632/vhwc42cmmy.1.

## Ethics statement

The studies involving human participants were reviewed and approved by National Bioethics Committee Iceland. The patients/participants provided their written informed consent to participate in this study.

## Author contributions

YH and RPÓ: conceptualization and supervision. YH (for EEG) and RPÓ (for psychological tests): methodology. YH: software, resources, data curation, visualization, project administration, and funding acquisition. YH, STJ, and AHH: validation. YH and AH: formal analysis. STJ and AHH: investigation. YH and STJ: writing—original draft preparation. RPÓ, STJ, and AHH: writing—review and editing. All authors have read and agreed to the published version of the manuscript.

## Funding

This study was supported by the Research Fund of University of Akureyri (RHA, R1916) and Icelandic Research Fund (Grant No. 228739-051).

## Conflict of interest

The authors declare that the research was conducted in the absence of any commercial or financial relationships that could be construed as a potential conflict of interest.

## Publisher's note

All claims expressed in this article are solely those of the authors and do not necessarily represent those of their affiliated organizations, or those of the publisher, the editors and the reviewers. Any product that may be evaluated in this article, or claim that may be made by its manufacturer, is not guaranteed or endorsed by the publisher.
